# 4499 A look at motivation and income level for families in obesity treatment

**DOI:** 10.1017/cts.2020.366

**Published:** 2020-07-29

**Authors:** Melissa Ramel, Denise Wilfley, Rachel Tabak

**Affiliations:** 1 Washington University in St. Louis

## Abstract

OBJECTIVES/GOALS: An evidence-based approach for childhood obesity is family-based treatment (FBT). Research supports that motivation and income level may impact treatment success; however, the relationship between the two is understudied. Therefore, the objective of this study was to examine whether motivation for beginning FBT is associated with income levels. METHODS/STUDY POPULATION: 459 parent and child dyads from the PLAN (Pediatric, Learning, Activity, Nutrition) with Families multisite study were included in this study. PLAN consists of FBT through personalized health coaching over the course of two years, focusing on nutrition, physical activity, and parenting skills. Parent and child also attend height and weight assessments every 6 months in the study. Outcomes of the study include weight change and mastery of behavioral skills. Motivation and income level were provided by self-report at the beginning of the study. Motivation was based on a scale from 1-10 (1 = no motivation, 10 = high motivation). Income levels were grouped into one of three broader categories- low income ($80,000/year). RESULTS/ANTICIPATED RESULTS: The mean level of motivation for the parent was 8.76 and for the child was 7.87. There was a significant difference in the mean level of motivation for the child and parent, t = 7.73, p = < .001. Post-hoc multiple comparisons using Tukey’s HSD test indicated that children in the high-income group had lower levels of motivation (M = 7.29, SD = 2.07) compared to children in the middle (M = 8.18) and low (M = 8.70) income groups. Level of motivation did not differ for children in the middle and low-income groups. Finally, parent motivation level did not differ significantly by income group. While there were significant differences between parent and child motivation levels, the motivation remained high for both groups. DISCUSSION/SIGNIFICANCE OF IMPACT: The data suggests a significant difference in mean child motivation and income level. Child’s high motivation may be from the idea of participating in something new, a rare opportunity for low-income children. To improve the implementation and efficacy of FBT, further study into the relationship between motivation and income level should be done.

